# Research progress on the molecular mechanisms of PD-1 and LAG-3 synergy in regulating T cell exhaustion and immunotherapy

**DOI:** 10.1080/07853890.2026.2643042

**Published:** 2026-03-17

**Authors:** Mei Li, Danxia Duan, Chengyuan Liu, Qiuxia Xiong

**Affiliations:** a Department of Medical Laboratory, The First Affiliated Hospital of Kunming Medical University, Kunming, China; b Yunnan Provincial Key Laboratory of Laboratory Medicine, Kunming, China; c Yunnan Provincial Clinical Research Center for Medical Laboratory Medicine, Kunming, China

**Keywords:** Tumor immunity, tumor immunology, T cell exhaustion PD-1 LAG-3, tumor immunotherapy

## Abstract

**Background:**

PD-1 and LAG-3 are immune checkpoint molecules frequently co-expressed in the tumor microenvironment, where they synergistically drive T-cell exhaustion and immune escape. Dual blockade of these pathways represents a promising strategy to overcome immunotherapy resistance.

**Methods:**

This review systematically synthesizes mechanistic studies on PD-1/LAG-3 synergy and summarizes preclinical and clinical advances in dual blockade therapies, with emphasis on bispecific antibodies and combination trial data.

**Results:**

PD-1 and LAG-3 cooperatively suppress T-cell function *via* complementary signaling pathways. Combined inhibition substantially restores antitumor immunity. A PD-1/LAG-3 bispecific antibody has been approved for melanoma, with numerous trials ongoing across other malignancies.

**Conclusion:**

Despite demonstrated efficacy, challenges including variable response rates, resistance mechanisms, and immune-related adverse events remain. Future efforts should prioritize biomarker identification and regimen optimization to enable precision application of dual blockade.

With the rapid advancement of cancer immunotherapy, combination strategies targeting immune checkpoints have emerged as a prominent research frontier in the field. Among these, the synergistic interaction between programmed death protein-1 (PD-1) and lymphocyte activation gene-3 (LAG-3) has garnered significant attention and is regarded as a critical breakthrough for enhancing the efficacy of immunotherapeutic approaches. PD-1 is expressed on the surface of antigen-stimulated T cells, where its signaling attenuates downstream pathways of the T cell receptor, thereby suppressing T cell activation, proliferation, and cytokine secretion. Under physiological conditions, PD-1 expression decreases rapidly after antigen clearance. However, in settings of persistent antigen exposure such as tumors, PD-1 remains highly expressed, ultimately leading to T cell exhaustion [1]. LAG-3, a key inhibitory immunoregulatory molecule, plays a crucial role in adaptive immune responses by modulating signal transduction between T lymphocytes and antigen-presenting cells [2]. Studies have shown that blocking LAG-3 not only restores the cytotoxic function of exhausted T cells but also attenuates the immunosuppressive activity of regulatory T cells, thereby enhancing antitumor immunity. Notably, multiple lines of evidence indicate that PD-1 and LAG-3 are frequently co-expressed in the tumor immune microenvironment and collaboratively mediate immune evasion [3]. Therefore, systematically elucidating the synergistic mechanisms through which these two molecules drive T cell exhaustion holds substantial theoretical significance and clinical relevance for optimizing existing immune checkpoint inhibitor therapies and developing novel combination strategies. This review aims to summarize recent research progress in this area, with the goal of providing academic insights to strengthen antitumor immune responses and improve clinical outcomes.

## 1. Characteristics of T cell exhaustion

**Figure 1.**
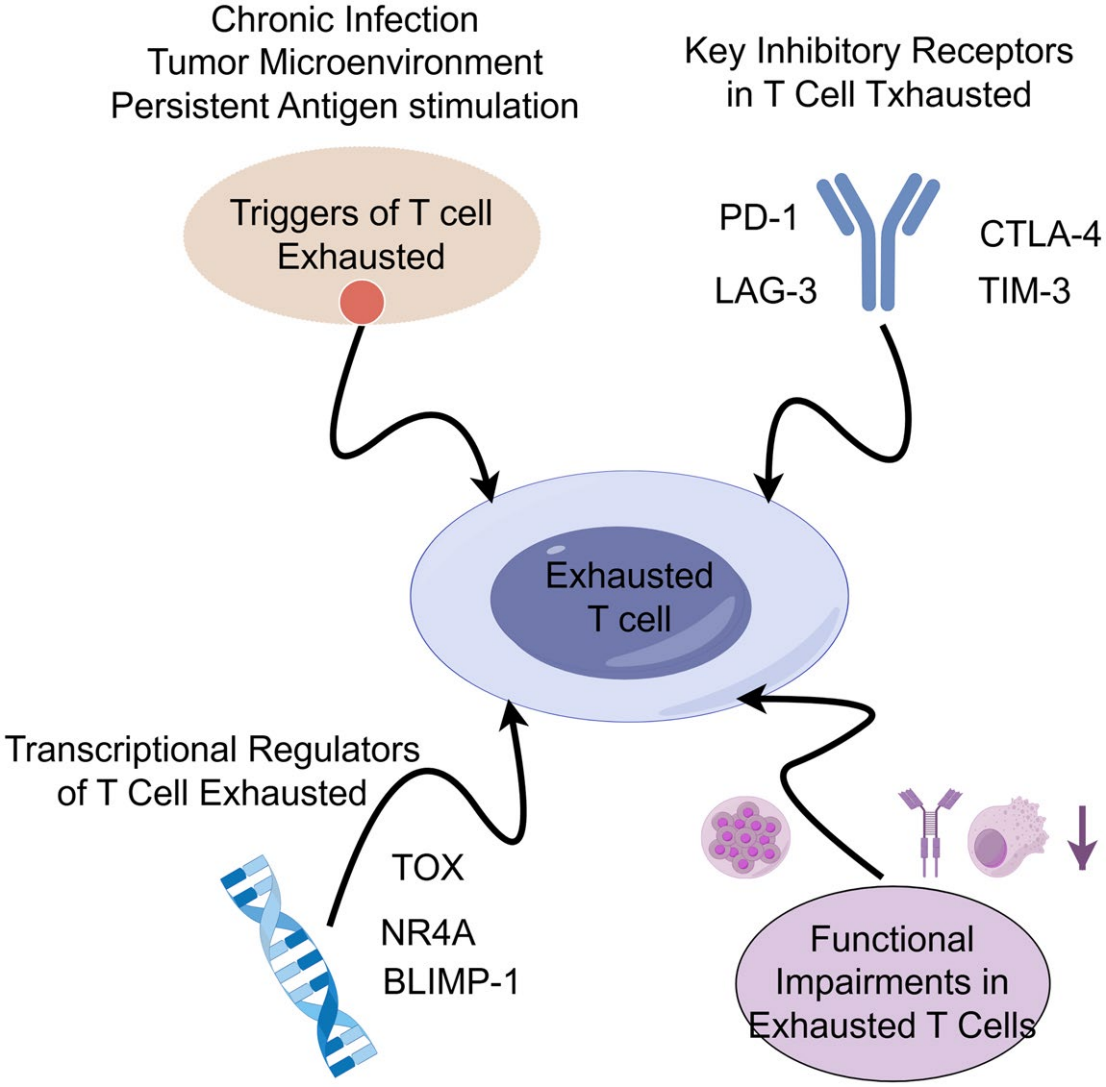
Key mechanisms of T cell exhaustion.

T cell exhaustion refers to a state of T cell dysfunction, characterized primarily by impaired responsiveness to infected or malignant cells [4]. During chronic infections, persistent antigen stimulation leads to prolonged activation of antigen-specific CD8^+^ T cells, resulting in functional impairment and an inability to effectively eliminate virus-infected cells [5]. In the tumor microenvironment, prolonged antigen exposure similarly induces a progressive decline in T cell functionality, marked by the loss of key effector capacities such as the production of interleukin-2 (IL-2), tumor necrosis factor-α (TNF-α), and interferon-γ (IFN-γ) [6]. Although CD8^+^ T cells can infiltrate tumor tissues, they often fail to mount an effective antitumor response, which contributes to diminished tumor control and sustained tumor growth [4]. Additionally, exhausted T cells exhibit impaired proliferative capacity upon antigen re-encounter, leading to insufficient strength and durability of immune responses. This progressive functional decline severely compromises the ability of CD8^+^ T cells to control chronic infections and malignancies.A hallmark of T cell exhaustion is the upregulation of inhibitory receptors, which is commonly observed in settings of chronic antigen exposure such as persistent infections and the tumor microenvironment [4]. Prolonged antigen stimulation drives T cells toward a state of progressive dysfunction, accompanied by elevated expression of inhibitory receptors such as PD-1, LAG-3, and CTLA-4. These receptors, upon interaction with their respective ligands, suppress T cell activation, proliferation, and effector functions. Moreover, sustained inhibitory signaling not only attenuates T cell–mediated immunity but also promotes immune evasion, thereby facilitating the persistence of infections and tumor progression [7]. Taken together, T cell exhaustion represents a multistage process of functional decline driven by chronic antigen exposure and associated with the upregulation of inhibitory receptors, significantly undermining immune control over chronic infections and cancer (Figure 1).

T cell exhaustion is a multifactorial process of immune dysfunction. It is primarily triggered by chronic infection, tumor microenvironment, and persistent antigen stimulation. During this process, exhausted T cells exhibit upregulated expression of inhibitory receptors (e.g. PD-1, CTLA-4, TIM-3, LAG-3) and are regulated by key transcriptional factors (e.g. TOX, NR4A, BLIMP-1), ultimately leading to functional impairments including diminished proliferative capacity, reduced cytokine production, and impaired cytotoxic activity.

## 2. Biological characteristics of PD-1 and LAG-3

### 2.1. Biological characteristics of PD-1

Programmed death receptor-1 (PD-1, CD279) is a 55-kD type I transmembrane protein belonging to the CD28 family. Its structure includes an extracellular immunoglobulin variable (IgV) domain, a transmembrane region, and an intracellular segment containing an immunoreceptor tyrosine-based inhibitory motif (ITIM) and an immunoreceptor tyrosine-based switch motif (ITSM), with ITSM activation being critical for PD-1‑mediated inhibitory function [8]. The extracellular domain of PD-1 contains multiple phosphorylation sites, including Y248, Y223, and Y201, which modulate its immunoregulatory activity [8]. PD-1 is primarily expressed on activated T cells, B cells, myeloid cells (such as dendritic cells and macrophages), natural killer cells, regulatory T cells (Tregs), and various tumor-infiltrating lymphocytes (TILs), with its expression regulated by T cell receptor activation and cytokine signaling [9]^.^ PD-1 has two ligands: PD-L1 (B7‑H1/CD274) and PD-L2 (B7‑DC/CD273), both of which are inhibitory. PD‑L1 is widely expressed on diverse cell types, including T cells, B cells, dendritic cells, macrophages, vascular endothelial cells, and pancreatic β‑cells, whereas PD‑L2 expression is more restricted and mainly observed on macrophages and dendritic cells. The PD-1/PD‑L1 pathway suppresses T cell proliferation, activation, and survival by recruiting SHP‑2 phosphatase to the intracellular domain of PD-1, thereby inhibiting PI3K‑AKT and MAPK signaling pathways in T cells. This mechanism represents a key process in tumor immune evasion [10]. While physiologically important for maintaining immune tolerance, it also drives T cell exhaustion within the tumor microenvironment and facilitates immune escape. It is precisely this dual role that makes PD-1 a crucial target for cancer immunotherapy, as its blockade can effectively restore the antitumor activity of T cells.

### 2.2. Biological characteristics of LAG-3

Lymphocyte activation gene 3 (LAG-3), also known as CD233, is a novel immune checkpoint receptor. It is a type I transmembrane protein consisting of an extracellular region, a transmembrane domain, and an intracellular segment. The extracellular portion comprises four immunoglobulin-like domains (D1–D4), which primarily mediate ligand binding, while the intracellular ‘KIEELE’ motif plays a crucial role in sustaining LAG-3 function [11,12]^.^ Studies have demonstrated that LAG-3 suppresses T cell activation by interacting with MHC-II as well as with other ligands, including fibrinogen-like protein 1 (FGL-1), α-synuclein fibrils (α-syn), galectin-3 (GAL-3), and liver sinusoidal endothelial cell lectin (LSECtin). These interactions impair the ability of T cells to produce cytokines and proliferate, leading to the inhibition of immune cell functions [13]^.^ One of the principal functions of LAG-3 is to negatively regulate MHC II-mediated T cell activation. LAG-3 shares approximately 20% sequence homology with CD4 and, similarly to CD4, can associate with the αβ T cell receptor (TCR) complex on the cell surface. However, unlike CD4, which enhances TCR signaling, LAG-3 binds to MHC-II concurrently with TCR engagement to antigen–MHC-II complexes on antigen-presenting cells. This interaction disrupts co-receptor–Lck association, thereby attenuating TCR signal transduction [14]. Consequently, the surface distribution and clustering of LAG-3 can modulate its regulatory activity.Moreover, the biology of LAG-3 is complex, as multiple ligands beyond MHC-II have been identified, including FGL1, LSECtin, Galectin-3, and α-synuclein fibrils. Although LAG-3 expression on T cells is typically induced in a TCR signaling-dependent manner, its presence and activity on immune cells lacking TCR expression—such as natural killer cells, B cells, and plasmacytoid dendritic cells—suggest that LAG-3 may exhibit diverse functional roles, likely mediated through interactions with distinct ligands [15].

## 3. Synergistic regulation mechanisms of PD-1 and LAG-3

### 3.1. Synergy at the signaling pathway level

T cell activation is a crucial step in the immune response, requiring coordinated interactions among multiple extracellular signaling stimuli. Among these, the T cell receptor (TCR)-mediated signaling pathway serves as a central axis. TCR initiates T cell activation by recognizing antigenic peptides presented by major histocompatibility complex (MHC) molecules on antigen-presenting cells (APCs), playing a vital role in processes such as T cell exhaustion and immune suppression [16]^.^ Programmed cell death protein 1 (PD-1) recruits Src homology 2 (SH2) domain-containing tyrosine phosphatase SHP-2 through its immunoreceptor tyrosine-based switch motif (ITSM) located in the cytoplasmic tail, thereby inhibiting the PI3K/Akt and MAPK signaling pathways. This suppression reduces T cell proliferation, cytokine secretion, and TCR signaling, ultimately impairing antigen recognition and activation of T cells [9]. On the other hand, lymphocyte activation gene-3 (LAG-3) competitively binds to MHC class II molecules, disrupting CD4–MHC II interactions and attenuating TCR signaling. LAG-3 also recruits SHP-1 and SHP-2 phosphatases to inhibit downstream signaling cascades and decrease the production of cytokines such as IFN-γ and IL-2 [17]^.^ Recent studies indicate that PD-1 and LAG-3 exhibit functional synergy in suppressing T cell activation signals, leading to impaired T cell function and exhaustion, thereby compromising antitumor and antiviral capabilities. Mechanistically, PD-1 primarily inhibits T cell proliferation, whereas LAG-3 predominantly suppresses effector functions. This complementary inhibition prevents T cells from expanding or exerting cytotoxic effects, ultimately contributing to T cell exhaustion [18]. Although PD-1 and LAG-3 operate through distinct signaling pathways, their concurrent inhibition more effectively suppresses T cell-mediated antitumor and antiviral responses. Combined checkpoint blockade of PD-1 and LAG-3 has been shown to significantly enhance T cell-mediated antitumor activity [19]^.^ For instance, clinical trials have demonstrated that dual therapy with relatlimab and nivolumab is both safe and effective, enhancing CD8^+^ TCR signaling, lowering the activation threshold, and reshaping CD8^+^ T cell differentiation to augment cytotoxicity while partially preserving exhaustion-associated features. Furthermore, Andrews et al. [20] observed in melanoma patients that nivolumab (an anti-PD-1 antibody) promotes clonal expansion of exhausted (Tex) cells, whereas relatlimab (an anti-LAG-3 antibody) enhances TCR signaling and sustains the terminal Tex cell population. Their combined administration results in stronger TCR signal transduction and enrichment of Tex cells, thereby improving effector functions. These findings further underscore the role of LAG-3 in regulating Tex cell development *via* the TOX–KLR axis. Combined deficiency of LAG-3 and PD-1 enhances Tex cell proliferation and effector functions, yielding synergistic antitumor efficacy.

### 3.2. Synergy at the cellular level

The synergistic inhibition of the aforementioned signaling pathways is ultimately reflected at the level of cellular phenotype and function. PD-1 is primarily expressed on activated T cells, and its expression is significantly upregulated upon antigen stimulation. Interaction between PD-1 and its ligands PD-L1 and PD-L2 suppresses receptor-mediated signaling, thereby inhibiting T cell activation, proliferation, and cytokine production. Following antigen clearance, PD-1 expression typically decreases rapidly; however, persistent antigen exposure sustains PD-1 expression, leading to T cell exhaustion [21]. Similarly, LAG-3 expression is also induced on activated T cells, where it binds specific ligands to inhibit T cell signal transduction [22]. Evidence indicates that PD-1 and LAG-3 jointly drive T cells toward an exhausted phenotype, thereby limiting their effector functions and ultimately impairing antitumor immune responses [23]^.^ Ngiow et al. [7] reported that PD-1 plays a critical role in limiting the proliferation of exhausted CD8^+^ T cells. Furthermore, LAG-3 selectively suppresses the effector functions of exhausted CD8^+^ T cells, including cytokine secretion and cytotoxicity. Notably, PD-1 and LAG-3 are highly co‑expressed on both CD4^+^ and CD8^+^ T cells in the tumor microenvironment and during chronic infections. In patients with diffuse large B‑cell lymphoma (DLBCL), surface expression of PD-1 on peripheral CD4^+^ T cells did not differ significantly from that in healthy controls (*p* > 0.05), whereas LAG-3 expression showed statistically significant differences (*p* < 0.05). In contrast, both PD-1 and LAG-3 expression on CD8^+^ T cells of DLBCL patients differed markedly from controls (*p* < 0.001) [24]. Further studies revealed elevated levels of PD-1 and LAG-3 on CD4^+^ and CD8^+^ tumor-infiltrating lymphocytes (TILs), and their expression levels showed a positive correlation. Simultaneous blockade of PD-1 and LAG-3 co-expression on TILs synergistically enhanced the antitumor response of CD8^+^ T cells [24]. These findings underscore that co-expression of PD-1 and LAG-3 at the cellular population level is a key characteristic driving T cell exhaustion and provide new insights into the cooperative regulatory mechanisms underlying this process.

### 3.3. Synergy at the molecular level

The co-expression and functional synergy of PD-1 and LAG-3 at the cellular level fundamentally arise from their coordinated regulation of downstream key transcriptional programs. Both PD-1 and LAG-3 are crucial immune checkpoint molecules that play central roles in inducing T cell exhaustion. Recent studies have shown that activation of PD-1 and LAG-3 signaling pathways collectively upregulates transcription factors such as TOX and BATF, thereby promoting T cell exhaustion [25]. Cillo et al. [26] demonstrated that dual checkpoint blockade exerts a unique biphasic regulatory effect: it simultaneously activates the cytotoxic function of CD8^+^ T cells while preserving exhaustion-associated features during differentiation. Notably, this treatment led to the detection of a novel subset of CD8^+^ TCR clones that retained transcriptional exhaustion signatures but exhibited enhanced IFN-γ responsiveness and activation capacity. Gene network analysis revealed synergistic interactions among transcription factors such as PRDM1, BATF, ETV7, and TOX in co-regulating cytotoxicity- and exhaustion-related gene programs.Furthermore, Hübbe et al. [27] demonstrated that PD-1 and LAG-3 cooperation promotes tumor immune evasion. It has been shown that their dual blockade is effective and relatively safe in treating advanced or metastatic solid tumors. In a mouse melanoma model, Andrews et al. [20] observed that CD8^+^ T cells deficient in both PD-1 and LAG-3 exhibited superior tumor clearance and prolonged survival. Transcriptomic analysis indicated that loss of these checkpoints resulted in enhanced TCR clonality, upregulation of effector-like genes, and interferon response genes. These transcriptional changes promoted IFN-γ secretion and enhanced anti-tumor activity.Collectively, these studies elucidate at the molecular level that PD-1 and LAG-3 synergistically drive CD8^+^ T cells toward exhaustion by co-regulating a core set of transcription factors (such as TOX), thereby restricting T cell proliferation and effector functions. This cooperative regulation of the transcriptional network represents a central hub linking upstream signal inhibition with downstream loss of cellular functional phenotypes.

## 4. Research advances in PD-1 and LAG-3 combination therapy

### 4.1. Research progress in PD‑1/LAG‑3 inhibitors

In recent years, antibody‑based drugs targeting PD‑1/PD‑L1 have achieved remarkable progress in clinical oncology [28]. However, due to the limited response rates of monotherapy, researchers have begun exploring combination strategies that block multiple immune checkpoints simultaneously. Among these, the co‑inhibition of PD‑1 and LAG‑3 has demonstrated clinical potential superior to single‑agent approaches [29,30]. At present, in addition to the approved fixed‑dose combination therapy (Relatlimab + Nivolumab, marketed as Opdualag^®^) and several monoclonal antibodies in clinical development (e.g. Fianlimab by Regeneron), bispecific antibodies (BsAbs) have emerged as a major research direction in this field.Several bispecific antibodies targeting LAG‑3 have entered the research and development pipeline, primarily including LAG‑3/PD‑1, LAG‑3/PD‑L1, LAG‑3/CTLA‑4, and LAG‑3/TIGIT formats. Among these, tebotelimab (MGD013), as the first LAG‑3/PD‑1 bispecific antibody to enter clinical trials, can simultaneously bind both PD‑1 and LAG‑3 with high affinity, targeting T cells that co‑express these proteins. Studies show that tebotelimab treatment significantly enhances cytokine secretion by T cells upon antigen re‑stimulation, outperforming blockade of either pathway alone. It effectively elevates serum IFN‑γ levels, restores CD8^+^ T‑cell function, and increases the expression of cytotoxicity‑related markers [15]. Another bispecific antibody, CB213, constructed from human‑derived nanobodies with an asymmetric 2:1 binding format, has demonstrated the ability to suppress tumor growth and increase the number of antigen‑specific CD8^+^ T cells in preclinical studies [31]. Moreover, RO7247669, a bispecific molecule targeting both PD‑1 and LAG‑3, selectively activates PD‑1^+^/LAG‑3^+^ T cells and induces antitumor immune responses. Its safety and efficacy are currently being evaluated in multiple clinical trials [32]^.^ Another noteworthy PD‑1/LAG‑3 bispecific antibody, YG‑003D3, has shown potential to overcome PD‑(L)1 resistance in preclinical studies. Compared with anti‑PD‑1 monotherapy, anti‑LAG‑3 monotherapy, or their combination, YG‑003D3 exhibited superior antitumor activity in both *in vitro* and *in vivo* models [33]. This antibody effectively activates immune cells, promotes cytokine release, and demonstrates enhanced performance in human peripheral blood mononuclear cell (PBMC) binding assays compared with monoclonal antibody combinations. In PD‑1/LAG‑3 double‑knockout mouse models, YG‑003D3 significantly inhibited tumor growth and increased the number of tumor‑killing T cells, highlighting its therapeutic potential as a novel bispecific antibody [29]^.^ These advances indicate that next‑generation PD‑1/LAG‑3 co‑blockade strategies, represented by bispecific antibodies, are providing new directions to overcome the current limitations of immunotherapy.

### 4.2. Clinical research progress of PD‑1/LAG‑3 combination therapy

The synergistic inhibitory effect of immune checkpoint molecules PD‑1 and LAG‑3 in T‑cell exhaustion provides a theoretical basis for the clinical translation of combination blockade strategies. In recent years, PD‑1/LAG‑3 combination therapy has rapidly evolved from mechanistic research to clinical practice, emerging as an important direction to overcome immunotherapy resistance. Multiple therapeutic strategies have been developed, including fixed‑dose antibody combinations and bispecific antibodies, which have shown encouraging efficacy in numerous clinical studies.Key clinical trials have demonstrated breakthrough progress in PD‑1/LAG‑3 combination therapy. In metastatic melanoma, the phase III RELATIVITY‑047 study confirmed that the fixed‑dose combination of relatlimab (anti‑LAG‑3 antibody) plus nivolumab (anti‑PD‑1 antibody) significantly prolonged median progression‑free survival compared with nivolumab monotherapy (10.1 months vs. 4.6 months). This combination received FDA approval in 2022, becoming the first globally approved LAG‑3‑targeted therapy. Subsequent studies have further expanded its application scope: in neoadjuvant treatment of non‑small cell lung cancer (NSCLC), it achieved a 95% R0 resection rate and 93% 12‑month disease‑free survival; in metastatic head and neck squamous cell carcinoma, the objective response rate reached 29.7% with a complete response rate of 13.5%; and significant survival benefits were also observed in MSI‑H/dMMR or PD‑L1‑positive colorectal cancer [34–37]. In addition, other anti‑LAG‑3 monoclonal antibodies (e.g. fianlimab, LAG525) combined with PD‑1/PD‑L1 inhibitors have shown positive signals in phase II studies across various solid tumors, including PD‑1‑refractory melanoma. Meanwhile, bispecific antibodies, as a next‑generation therapeutic strategy, have also demonstrated significant potential. Studies indicate that PD‑1/LAG‑3 bispecific antibodies exhibit enhanced immunomodulatory functions and antitumor activity *in vitro* [33]. Among them, tebotelimab (MGD013) achieved a 34% tumor regression rate as monotherapy in patients with PD‑1‑resistant solid tumors and CAR‑T‑resistant lymphoma in a phase I study, and reached a 19% objective response rate when combined with anti‑HER2 therapy, with a manageable safety profile [38]. Other early‑phase clinical studies targeting NSCLC, hepatocellular carcinoma, gastric/gastroesophageal junction adenocarcinoma, triple‑negative breast cancer, and ovarian cancer have preliminarily confirmed the antitumor activity and safety of combination therapies, particularly observing disease remission in PD‑1/PD‑L1‑pretreated patients, suggesting potential for overcoming resistance. In hematologic malignancies, studies on diffuse large B‑cell lymphoma and classical Hodgkin lymphoma have also shown preliminary efficacy, especially in patients with T‑cell exhaustion and PD‑1/LAG‑3 co‑expression in the tumor microenvironment [34,39–46]^.^ Collectively, these findings indicate that the PD‑1/LAG‑3 dual‑blockade strategy holds broad clinical application prospects. It not only provides a new standard treatment option for advanced melanoma but also offers a promising breakthrough direction for addressing immunotherapy resistance in a variety of solid tumors and hematologic malignancies (Table 1).

**Table 1.** Summary of clinical trials of PD-1 and LAG-3 combination immune checkpoint inhibitor therapy.

ClinicalTrials.gov ID	Drug Name	Target	Disease Area	Phase	Sponsor	Study Period	Author
NCT03470922	nivolumab + relatlimab	PD-1、LAG-3	Untreated metastatic or unresectable melanoma	II/III	Bristol Myers Squibb(BMS)	2022-01-06	Lipson EJ et al. [37]
NCT03743766	nivolumab + relatlimab	PD-1、LAG-3	Patients with Metastatic Melanoma	II	Bristol Myers Squibb(BMS)	2019-03	Rush Eet al. [39]
RELATIVITY-060	nivolumab + relatlimab	PD-1、LAG-3	Untreated advanced gastric cancer (GC) or gastroesophageal junction cancer (GEJC) patients	II	Bristol Myers Squibb(BMS)	2018-10-16	Hegewisch-Becker S et al. [40]
NCT03642067	nivolumab + relatlimab	PD-1、LAG-3	Previously treated metastatic pMMR colorectal cancer patients	II	Bristol Myers Squibb(BMS)	2019–02	Christenson ES et al. [41]
NCT02060188	nivolumab + relatlimab	PD-1、LAG-3	Previously treated MSI-H/dMMR metastatic CRC patients	II	MJO in collaboration with Bristol Myers Squibb	2017-05	Overman MJ et al. [42]
NCT02061761	nivolumab + relatlimab	PD-1、LAG-3	Advanced relapsed/refractory B-cell malignancies	I/II	Bristol Myers Squibb(BMS)	2014-03-12	Gopal AK et al. [43]
NCT02996110	nivolumab + relatlimab	PD-1、LAG-3	Advanced renal cell carcinoma (aRCC) patients	II	Bristol Myers Squibb (BMS) and Ono Pharmaceutical Company Ltd	2017-02-02	Choueiri TK et al. [44]
NCT03026140	nivolumab + relatlimab	PD-1、LAG-3	Locally advanced mismatch repair-deficient colon cancer patients	II	Bristol Myers Squibb(BMS)	2022-12-15	de Gooyer PGM et al. [45]
NCT04205552	nivolumab + relatlimab	PD-1、LAG-3	Previously treated resectable non-small cell lung cancer patients	II 期	Bristol Myers Squibb(BMS)	2020-03-04	Schuler M et al. [34]
NCT03044613	nivolumab + relatlimab	PD-1、LAG-3	Resectable II/III stage gastroesophageal cancer patients	Ib	Bristol Myers Squibb (BMS), AstraZeneca	2017-08	Kelly RJ et al. [46]

## 5. Challenges of combination therapy

### 5.1. Limited therapeutic response and complex resistance mechanisms

The significant heterogeneity of tumors, individual variations in immune status among patients, and the complexity of the tumor microenvironment are central reasons why some patients fail to respond or exhibit only transient responses to combination therapy. Even when initially effective, tumor cells can adaptively develop immune escape mechanisms [47]. These primarily include: (1) Intrinsic tumor mechanisms: such as loss or mutation of antigen presentation machinery (MHC class I), preventing T cells from recognizing their targets [48]; inactivation of interferon‑γ signaling pathways (e.g. JAK1/2, STAT1), leading to tumor cell resistance to immune attack [49]; and enhancement of pro‑survival signals such as PTEN loss/PI3K pathway activation [50]. (2) Tumor microenvironment remodeling: Beyond PD‑1 and LAG‑3, the microenvironment often exhibits co‑expression of multiple inhibitory checkpoint molecules (e.g. TIM‑3, TIGIT, VISTA), forming a ‘complementary escape network’ where dual blockade alone may be insufficient to fully restore T cell function [51,52]. Furthermore, the enrichment of regulatory T cells (Tregs) and myeloid‑derived suppressor cells (MDSCs), along with sustained secretion of immunosuppressive factors (e.g. TGF‑β, IL‑10), establishes a profound immunosuppressive barrier [53]. (3) Physical and metabolic barriers: Structural and functional abnormalities of tumor vasculature severely impair T‑cell infiltration and distribution within tumors, while metabolic stressors in the microenvironment—such as hypoxia, lactate accumulation, and nutrient competition—directly suppress the survival and function of infiltrating T cells. Collectively, these factors undermine the efficacy of combination therapy and may contribute to acquired resistance [54].

### 5.2. Immune‑related adverse events and challenges in optimizing treatment strategies

PD‑1 inhibitor monotherapy is already associated with a spectrum of immune‑related adverse events (irAEs). Theoretically, combined blockade of LAG‑3 may further release immune system ‘brakes,’ potentially increasing the incidence and severity of irAEs [55]. Clinical data support this concern: for instance, in melanoma combination therapy, the incidence of grade 3–4 treatment‑related adverse events is significantly higher than with PD‑1 monotherapy. This poses greater challenges for long‑term patient tolerance and clinical toxicity management [56]. Beyond common irAEs (e.g. rash, colitis, pneumonitis, hepatitis, and endocrine disorders), it remains essential to monitor whether combination therapy may induce novel or unique toxicity profiles (such as potential neurological or cardiac effects) in larger‑scale, longer‑follow‑up real‑world studies. Excessive immune activation can target any normal tissue, and long‑term safety data are still pending [57]^.^ Currently, no unified standards exist for the optimal dosing, treatment duration, or sequencing of PD‑1/LAG‑3 co‑blockade. Balancing efficacy and toxicity to maximize the therapeutic window remains a critical issue in clinical practice. Moreover, while the potential for combining this regimen with other modalities (e.g. chemotherapy, radiotherapy, targeted therapy, or other immunotherapies) is substantial, designing rational combination strategies and determining optimal administration sequences to avoid additive toxicity and achieve true synergy will require extensive preclinical mechanistic exploration and meticulously designed clinical trials [58].

## 6. Summary and future perspectives

In recent years, programmed cell death protein‑1 (PD‑1) and lymphocyte activation gene‑3 (LAG‑3), as key immune checkpoint molecules, have been increasingly recognized for their synergistic role in regulating T‑cell exhaustion. Studies demonstrate that PD‑1 and LAG‑3 collectively impair T‑cell activation, proliferation, and effector functions through distinct signaling pathways, thereby promoting an exhausted phenotype. Their co‑expression is notably prominent in chronic infections and the tumor microenvironment, and combined blockade of PD‑1 and LAG‑3 has demonstrated significant synergistic antitumor effects in preclinical models. Currently, several clinical trials have confirmed the efficacy of PD‑1/LAG‑3 bispecific antibodies in malignancies such as melanoma, providing a new strategy to overcome resistance to immunotherapy.Nevertheless, combination therapy still faces limitations, including restricted therapeutic responses, complex resistance mechanisms, immune‑related adverse events, and challenges in optimizing treatment strategies. Future research should further elucidate the precise molecular network underlying PD‑1/LAG‑3 synergy, with particular focus on the regulatory roles of downstream epigenetic modifications and metabolic reprogramming. Additionally, exploring their interactions with other immune checkpoints (e.g. TIM‑3, TIGIT) and developing combination strategies with emerging therapeutic modalities—such as CAR‑T cell therapy and cancer vaccines—will contribute to the refinement of treatment regimens. Furthermore, the identification of individualized biomarkers (e.g. LAG‑3 ligand expression levels) and in‑depth investigation of resistance mechanisms will be crucial for advancing the clinical translation of this approach.

## Data Availability

There is no new data generated during the study.
